# Common Variable Immunodeficiency-Associated Cancers: The Role of Clinical Phenotypes, Immunological and Genetic Factors

**DOI:** 10.3389/fimmu.2022.742530

**Published:** 2022-02-17

**Authors:** Luzia Bruns, Victoria Panagiota, Sandra von Hardenberg, Gunnar Schmidt, Ignatius Ryan Adriawan, Eleni Sogka, Stefanie Hirsch, Gerrit Ahrenstorf, Torsten Witte, Reinhold Ernst Schmidt, Faranaz Atschekzei, Georgios Sogkas

**Affiliations:** ^1^ Department of Rheumatology and Immunology, Hannover Medical School, Hanover, Germany; ^2^ Department of Hematology, Hemostasis, Oncology, and Stem Cell Transplantation, Hannover Medical School, Hannover, Germany; ^3^ Department of Human Genetics, Hannover Medical School, Hanover, Germany; ^4^ Department of Medical Oncology, Papageorgiou Hospital, Aristotle University of Thessaloniki, Thessaloniki, Greece; ^5^ Hannover Medical School, Cluster of Excellence RESIST (EXC 2155), Hanover, Germany

**Keywords:** CVID, cancer, CTLA-4, NF-κB1 (NF-kappaB1), cancer immune surveillance

## Abstract

**Objective:**

The aim of this study was to investigate the prevalence of cancer and associating clinical, immunological, and genetic factors in a German cohort of patients with common variable immunodeficiency (CVID).

**Methods:**

In this retrospective monocenter cohort study, we estimated the standardized incidence ratio (SIR) for different forms of cancer diagnosed in CVID patients. Furthermore, we evaluated the likely association of infectious and non-infectious CVID-related phenotypes with the diagnosis of cancer by calculation of the odds ratio. The genetic background of CVID in patients with cancer was evaluated with sequential targeted next-generation sequencing (tNGS) and whole-exome sequencing (WES). Patients’ family history and WES data were evaluated for genetic predisposition to cancer.

**Results:**

A total of 27/219 patients (12.3%) were diagnosed with at least one type of cancer. Most common types of cancer were gastric cancer (SIR: 16.5), non-melanoma skin cancer (NMSC) (SIR: 12.7), and non-Hodgkin lymphoma (NHL) (SIR: 12.2). Immune dysregulation manifesting as arthritis, atrophic gastritis, or interstitial lung disease (ILD) was associated with the diagnosis of cancer. Furthermore, diagnosis of NMSC associated with the diagnosis of an alternative type of cancer. Studied immunological parameters did not display any significant difference between patients with cancer and those without. tNGS and/or WES yielded a definite or likely genetic diagnosis in 11.1% of CVID patients with cancer. Based on identified variants in cancer-associated genes, the types of diagnosed cancers, and family history data, 14.3% of studied patients may have a likely genetic susceptibility to cancer, falling under a known hereditary cancer syndrome.

**Conclusions:**

Gastric cancer, NMSC, and NHL are the most frequent CVID-associated types of cancer. Manifestations of immune dysregulation, such as arthritis and ILD, were identified as risk factors of malignancy in CVID, whereas studied immunological parameters or the identification of a monogenic form of CVID appears to have a limited role in the evaluation of cancer risk in CVID.

## Introduction

Common variable immunodeficiency (CVID) is the most common symptomatic primary immunodeficiency, comprising a heterogeneous group of disorders, which all associate with primary antibody production failure (1). Besides unusual infections, non-infectious manifestations including autoimmunity, autoinflammation, and polyclonal lymphoproliferation can precede the onset of clinically evident immunodeficiency and dominate the phenotype of CVID (2). According to a recent meta-analysis, the prevalence of malignancy in CVID was 8.6% (3). Most common types of cancer, which also display a relatively higher incidence in CVID patients, are lymphomas and gastric cancer (3–5). In contrast, there appears to be no association of CVID with other common types of cancer (6), such as lung or prostate cancer.

Next-generation sequencing (NGS) has considerably advanced our understanding of the genetic background of primary immunodeficiency disorders (PIDs) and led to the identification of an increasing number of monogenic forms of CVID (7). Furthermore, genetic studies provided new insights into the pathogenesis of PID-associated manifestations, such as autoimmunity and polyclonal lymphoproliferation (8). The same holds true for PID-associated cancers, where PID-associated genetic variants can either directly confer susceptibility to cancer or indirectly support carcinogenesis by causing genetic instability, persistent lymphoproliferation, and/or oncogenic infections (9, 10). Notable monogenic PIDs, whose genetic background directly predisposes to cancer, include the activated phosphoinositide 3-kinase δ syndrome (APDS), nuclear factor kappa B subunit 1 (NF-κB1) insufficiency, and Signal transducer and activator of transcription 3 (STAT3) gain-of-function syndrome (11–13). Evidence for this stems from the detection of somatic mutations in *PIK3CD*, *NFKB1*, and *STAT3* in human cancer genomes and the identification of their role in carcinogenesis (14–17).

The association of CVID with malignancy has not been integrated into clinical practice, and patients are not routinely offered screening for prevention and early diagnosis of cancer. The identification of the prevalence and relative incidence of cancer in CVID could raise awareness of malignancy as a major manifestation of CVID. Furthermore, the characterization of comorbidities, immunological abnormalities, or genetic factors associating with the diagnosis of cancer in CVID could improve our understanding of carcinogenesis in CVID and eventually contribute to the development of effective cancer screening. This monocentric retrospective study was designed to evaluate the clinical phenotypes, the immunological profiles, and the genetic factors associating with the diagnosis of cancer in CVID.

## Materials and Methods

### Study Cohort

This study included a total of 219 patients with CVID visiting the immunology outpatient clinics of the Department of Rheumatology and Immunology of the Hannover Medical School. Data were collected from 2015 to 2021. Following comment has been added: Diagnosis of CVID was based on the current European Society for Immunodeficiencies (ESID) diagnostic criteria [available at http://esid.org/Working-Parties/Registry/Diagnosis-criteria (18)] or the original ESID/Pan-American Group for Immunodeficiency (PAGID) (1999) criteria (available at https://esid.org/Education/Common-Variable-Immunodeficiency-CVI-diagnosis-criteria). Among those originally diagnosed on the basis of the ESID/PAGID criteria, this study included only patients with reduced immunoglobulin A (IgA) levels, poor antibody response to vaccines, or absent isohemagglutinins or low class-switched memory B cells and CD4^+^ T-cell counts >200/µl. Immunological and clinical data were obtained from patients’ medical files. Immunological data included serum immunoglobulin levels and counts of major lymphocyte subsets at diagnosis of CVID. Documented clinical data included clinical history of infections, bronchiectasis [computed tomography (CT)-confirmed], autoimmune cytopenias, such as autoimmune hemolytic anemia (AIHA), idiopathic thrombocytopenic purpura (ITP), organ-specific autoimmunity [including vitiligo, psoriasis, insulin-dependent diabetes mellitus (IDDM), thyroidopathies, atrophic gastritis, and arthritis], granulomatous disease, enteropathy, and malignancies. CVID-associated interstitial lung disease (ILD) was diagnosed based on typical CT scan findings in the absence of evidence for an infectious or alternative cause. Splenomegaly was defined as spleen enlargement ≥11 cm on palpation or ultrasound, including previous splenectomy of an enlarged spleen. Lymphadenopathy was detected on palpation, ultrasound, CT, or magnetic resonance scan. Granulomatous disease was defined as at least one biopsy-proven unexplained granuloma, excluding Crohn’s disease-associated granulomas. Enteropathy included all cases of biopsy-proven non-infectious inflammatory bowel disease (ulcerative colitis and Crohn’s disease) and intestinal hyperlymphocytosis (lymphocytic infiltration of the interepithelial mucous, the lamina propria, and/or the submucosa). Malignancies included hematologic and all other forms of cancer. Diagnosis and staging of cancer were made according to relevant contemporary German Cancer Society guidelines (available at https://www.leitlinienprogramm-onkologie.de/leitlinien/). Briefly, colon cancer and gastric cancer were diagnosed with gastrointestinal endoscopy coupled with biopsies. Breast cancer was diagnosed with standard imaging techniques after histologic confirmation. Lymphomas were diagnosed on the basis of imaging, including CT, MRI, and PET, as well as histological examination of tissue sections, lymph nodes, and/or bone marrow biopsies. The diagnosis of non-melanoma skin cancer (NMSC) was made clinically and confirmed histologically after tumor excision. Family history of all 27 patients diagnosed with cancer has been assessed for a potential hereditary cancer syndrome based on the European Society for Medical Oncology (ESMO) guidelines for hereditary cancer syndromes (https://www.esmo.org/guidelines/hereditary-syndromes). In particular, each patient’s family history has been retrospectively evaluated based on patients’ medical files for following red flags of hereditary cancer: 1) more than two affected close relatives (parents, children, siblings, grandparents, aunts/uncles, and first cousins) from the same family side with same or related cancers (i.e., two or more of the following cancers: breast, ovarian, prostate, pancreatic; two or more of the following cancers: colorectal, endometrial, ovarian, gastric, pancreatic; two or more of the following cancers: breast, thyroid, endometrial, colorectal, melanoma, kidney); 2) diagnosis of cancer before the age of 50 years; 3) rare forms of cancer (such as ovarian cancer, sarcomas, adrenocortical carcinoma, choroid plexus carcinoma); 4) Ashkenazi Jewish ancestry; and 5) consanguinity. In case of 20/27 studied patients, we were additionally able to take a structured family history during the current study, focusing on family history of cancer and all the abovementioned red flags of hereditary cancer.

All patients signed an informed consent form. This study was approved by the ethics review board of the Hannover Medical School Ethics Committee (ethics vote number: 5582; 8875_BO_K_2020).

### Next-Generation Sequencing

Blood samples were collected in the immunology outpatient clinics of the Department of Rheumatology and Immunology of the Hannover University School. Genomic DNA was extracted by QIAamp DNA Blood Midi Kit (Qiagen) and quantified by Qubit dsDNA BR Assay Kit (Thermo Fisher). All 27 patients diagnosed with cancer were sequenced by means of targeted next-generation sequencing (tNGS), which was performed as described previously (19). Briefly, a customized panel of genes associated with PIDs (19) was created with the help of Agilent’s web-based SureDesign application. DNA target enrichment was performed using Agilent’s HaloPlex Target Enrichment System for Illumina sequencing following the manufacturer’s instructions (Agilent’s user manual). Sequencing was performed on an Illumina MiSeq system using an Illumina v2 reagent kit following the manufacturer’s protocol. The FastQ files were aligned to the human reference genome (UCSC hg19, *GRCh37*) and analyzed using Agilent’s SureCall software as described previously (19) Whole-exome sequencing (WES) was performed on genomic DNA samples from 21/27 patients diagnosed with cancer as described previously (20). Briefly, the concentration and quality of the purified genomic DNA (gDNA) were determined with an Agilent Technologies 2100 Bioanalyzer (Agilent Technologies, Santa Clara, CA, USA). The DNA sequencing library consisted of 100 ng fragmented gDNA and was generated with Agilent SureSelectXT Reagent Kits v5 UTR (70 Mb) according to the manufacturer’s protocols (Illumina, San Diego, CA, USA). Libraries were sequenced on an Illumina HiSeq2500 platform using TruSeq SBS Kit v3-HS (200 cycles, paired end run) with an average of 12.5 × 106 reads per single exome (mean coverage: 50×). The GATK-Pipeline (GenomeAnalysisTK-1.7) was applied for read quality trimming, read alignment to reference (UCSC hg19, GRCh37), and quality trimmed variant calling. Variant annotation was performed using Gsvar software. Patient exomes were filtered for mutations in 490 genes associated with PIDs as well as 125 cancer-associated genes (**Supplementary Table 1**). Genetic variants in PID and cancer-associated genes were excluded if the allele frequencies in the general population were >1% in the Exome Aggregation Consortium database (ExAC) or the Genome Aggregation Database (gnomAD). Besides allele frequency, identified variants were selected according to the following criteria: variant annotation and potential functional effect using databases of variants [e.g., dbSNP, 1000 Genomes Project, Exome Aggregation Consortium (ExAC), gnomAD] and disease-causing variants [Human Gene Mutation Database (HGMD), Online Mendelian Inheritance in Man (OMIM)]. Furthermore, we kept nonsense variants, variants affecting splice site, frameshift, in-frame indels, start or stop codon changes, as well as missense variants that were predicted deleterious by having a combined annotation-dependent depletion (CADD) score ≥20 and a mutation significance cutoff (MSC) score below the CADD score (21, 22). Targeted sequencing findings are publicly available under following links: https://www.ncbi.nlm.nih.gov/bioproject/PRJNA750325/PRJNA750325.

### Expression of Cytotoxic T-Lymphocyte-Associated Protein 4

Whole blood was collected in ethylene diamine tetraacetic acid (EDTA) tubes, and peripheral blood mononuclear cell (PBMC) preparations have been performed as described previously (23). PBMCs were cryopreserved in freezing medium (heat-inactivated fetal calf serum (FCS), PAN-Biotech; 10% v/v dimethyl sulfoxide, Sigma) until use. Upon cell thawing, PBMCs were rested overnight in complete RPMI medium (at 37°C, 5% CO_2_) for recovery and collected in the morning for further processing. After washing once in fluorescence-activated cell sorting (FACS) buffer [phosphate buffered saline (PBS), 5% v/v FCS], the cells were stained with antibodies in the presence of Octagam 10% (Octapharma). PBMCs were stained with CD4-BV421 (clone RPA-T4, BD Biosciences), CD25-BV510 (clone 2A3, BD Biosciences-OptiBuild), and CD127-PE (clone A019D5, BioLegend) antibodies and Fixable Viability Dye eFluor780 (Thermo Fisher). Regulatory T cells (Tregs) (live CD4^+^ CD25^hi^ CD127^lo^) were sorted under aseptic condition in BD FACSAria Fusion cell sorter (Becton-Dickinson). Cell sorting typically yielded high enrichment (>90%) of Tregs. Sorted Tregs were stimulated with anti-CD3/CD28 beads (Dynabeads, Thermo Fisher) for 16 h. Cytotoxic T-lymphocyte-associated protein 4 (CTLA-4) expression was assessed in Cyto-Fast Fix/Perm (BioLegend)-permeabilized cells using a CTLA-4-APC antibody (BioLegend).

### Statistical Analysis

For statistical calculation, we used GraphPad prism 9 (GraphPad, La Jolla, USA). Descriptive statistics are reported as median and interquartile range (IQR) in case of continuous variables and as counts and percentages for dichotomous variables. Categorical variables were compared by the Fisher’s exact test. Differences between patients with and without cancer were evaluated with the Mann–Whitney test. Comparison of more than two groups was performed with the Kruskal–Wallis test. To correct for multiple testing, p values were adjusted for Benjamini–Hochberg false discovery rate (FDR). The p values were considered significant if they were lower than a threshold selected to control an FDR of 10%. To calculate standardized incidence ratios (SIRs), all patients were stratified by age and gender. Data on the reference population were derived from the 12th edition of the “Cancer in Germany” report by the Robert Koch Institute (23). The expected number of cases for each type of cancer in the studied patient cohort was calculated based on the age- and gender-specific incidence rate provided in the aforementioned report. SIR was calculated by dividing the actual number of cases in the studied patient cohort by the one in the reference population.

## Results

### Clinical Characterization of Common Variable Immunodeficiency Patients

Patients’ demographic data and characteristics are summarized in **Table 1**. Most patients had sporadic immunodeficiency. Besides recurrent infections, most patients had a history of at least one non-infectious CVID-associated manifestation (157/219, 71.7%). Those included benign lymphoproliferation, manifesting as lymphadenopathy and/or splenomegaly (107/219, 48.9%), autoimmune disease (82/219, 37.4%), or atopic disease (37/219, 16.9%). A total of 27 patients (12.3%) were diagnosed with at least one form of cancer. Their clinical characteristics, including cancer type and stage, are summarized in **Supplementary Table 2**. Median age of first diagnosis of cancer was 45 years (IQR: 37–58 years). In most cases, the first diagnosis of cancer followed the diagnosis of CVID (20/27, 74.1%). The median time from diagnosis of CVID to diagnosis of cancer was 4 years (IQR: 1–13 years). Furthermore, 7/219 patients (3.2%) were diagnosed with more than one form of cancer. Diagnosis of cancer was equally common in sporadic and familial cases of CVID (3/27 vs. 17/192; p = 0.72). At the time of data analysis, 5/27 patients were deceased, and one was lost to follow-up (**Supplementary Table 2**). Prevalence and SIR for different types of cancer are presented in **Table 2**. Gastric cancer followed by NMSC and non-Hodgkin lymphoma (NHL) appeared to be considerably more common in CVID, displaying the highest SIR values. Besides the higher prevalence of those three forms of cancer, all of them appear to be diagnosed considerably earlier in CVID than in the general population (**Supplementary Table 3**) (24). Interestingly, gastric cancer was diagnosed at an early stage and at a considerably younger age (median: 36.5 years, IQR: 35–69) than in the general population (24). Regarding NHL, similar to the general population (25), most CVID patients (5/6) developed B-cell lymphomas. Furthermore, all cases of breast cancer were early-stage hormone receptor (HR) positive and human epidermal growth factor receptor (HER-2) negative, which is the most prevalent breast cancer in the general population (26).

**Table 1:** Characteristics of studied patients (*N* = 219).

Median age at diagnosis of CVID^1^, years (IQR)	33 (21–45)
Male sex, no. (%)	91 (41.6)
Familial cases, no. (%)	20/219 (9.1)
History of parental consanguinity, no. (%)	2/219 (0.9)
Recurrent pneumonias^2^, no. (%)	148 (67.6)
Bronchiectasis, no. (%)	51 (23.3)
Recurrent gastrointestinal infections^2^, no. (%)	46 (21)
“Infections only” disease, no. (%)	62 (28.3)
Benign lymphoproliferation, no. (%)	103 (47)
Splenomegaly, no. (%)	22 (10)
Enteropathy, no. (%)	27 (12.3)
ILD, no. (%)	22 (10)
ITP, no. (%)	32 (14.6)
AIHA, no. (%)	13 (5.9)
Psoriasis, no. (%)	10 (4.6)
Vitiligo, no. (%)	8 (3.7)
Thyroidopathy, no. (%)	14 (6.4)
Atrophic gastritis, no. (%)	6 (2.7)
Arthritis, no. (%)	16 (7.3)
Atopic disease^3^, no. (%)	37 (16.9)
Granulomatous disease, no. (%)	25 (11.4)
Cancer, no. (%)	27 (12.3)
Immunoglobulin replacement, no. (%)	205 (93.6)
Immunosuppressive regimens, no. (%)	74 (33.8)

AIHA, autoimmune hemolytic anemia; CVID, common variable immunodeficiency; ILD, interstitial lung disease; IQR, interquartile range; ITP, immune thrombocytopenic purpura; no., number.

^1^Analysis based on 202/219 studied patients with known year of diagnosis.

^2^At least two documented pneumonias/gastrointestinal infections.

^3^Atopic dermatitis and/or allergic rhinitis and/or asthma.

**Table 2:** Prevalence and SIR for different types of cancer in a cohort of 219 CVID patients.

Cancer	Prevalence N (%)	SIR (95% CI)
Breast cancer	6 (2.74)	1.87 (0.6–4.36)
Lung cancer	1 (0.46)	0.69 (0.9–3.82)
NHL	6 (2.74)	12.2 (4.46–26.57)
Gastric cancer	6 (2.74)	16.54 (6.04–36.01)
Colorectal cancer	4 (1.83)	2.8 (0.75–7.18)
NMSC	8 (3.65)	12.74 (5.1–26.27)

CVID, common variable immunodeficiency; NMSC, non-melanoma skin cancer; NHL, non-Hodgkin lymphoma; SIR, standardized incidence ratio.

### Common Variable Immunodeficiency Manifestations Associating With Cancer

Considering all 219 patients, arthritis, atrophic gastritis, and ILD were the CVID-associated manifestations, which were significantly more common among patients with cancer (**Table 3**). Furthermore, NMSC was diagnosed in 5/7 with more than one type of cancer. Most of those 5 patients (4/5) had received no radiation therapy or chemotherapy prior to diagnosis of NMSC, which would result in increased risk for NMSC (27). NMSC, and in particular basal cell carcinoma, significantly associated with the diagnosis of an additional form of cancer [5/24 vs. 3/195; p < 0.005; odds ratio (OR): 16.84, IQR: 3.73–76.04]. In 3 out of 5 cases with a diagnosis of more than one form of cancer, including NMSC, diagnosis of NMSC preceded the diagnosis of an additional form of cancer. A subgroup analysis was performed for the relatively more common forms of cancer, i.e., NMSC, NHL, gastric cancer, and breast cancer. As reported in previous studies (4), CVID patients with gastric cancer were more commonly diagnosed with atrophic gastritis (p = 0.009; OR = 26.11, IQR: 3.67–186.4) and had a history of recurrent gastrointestinal infections (p = 0.0187; OR = 8.14, IQR: 1.44–45.98) or infection with *Helicobacter pylori* (p = 0.0256; OR = 12.81, IQR: 2.04–80.59). For the rest of the diagnosed forms of cancer, we identified no significant association with any of the studied CVID manifestations. Also treatment with immunoglobulin replacement or immunosuppressive agents did not associate with the diagnosis of cancer (**Table 4**).

**Table 3:** Association of infectious and non-infectious manifestations of CVID with cancer.

	Variable	At least one cancer (*N* = 27)	No cancer (*N* = 192)	OR (95% CI)	p^+^	*q*
immune dysregulation	AIHA	2	11	1.32 (0.28–6.29)	0.6654 (ns)	0.8555
	ITP	5	27	1.39 (0.48–3.98)	0.5611 (ns)	0.7769
	Arthritis	5	10	4.14 (1.3–13.2)	0.0246 (ns)	0.1476
	Atopic disease	6	31	1.48 (0.55–3.98)	0.4171 (ns)	0.7508
	Atrophic gastritis	4	2	16.52 (2.87–95.28)	0.0024 (**)	0.0432
	Enteropathy	5	22	1.76 (0.6–5.11)	0.3442 (ns)	0.7508
	Granulomatous disease	5	20	1.96 (0.67–5.73)	0.2064 (ns)	0.6192
	ILD	7	14	4.45 (1.61–12.32)	0.0070 (**)	0.0630
	Lymphadenopathy	15	88	1.48 (0.66–3.23)	0.4119 (ns)	0.7508
	Psoriasis	1	9	0.78 (0.1–6.43)	1.000 (ns)	1.0000
	Splenomegaly	4	18	1.68 (0.52–5.4)	0.4895 (ns)	0.7769
	Thyroidopathy	2	13	1.1 (0.23–5.17)	1.000 (ns)	1.0000
infectious manifestations	Bronchiectasis	5	42	0.81 (0.29–2.27)	0.8064 (ns)	0.9255
	Infections only disease	7	57	0.83 (0.33–2.1)	0.8227 (ns)	0.9255
	*H. pylori*	3	7	3.3 (0.8–13.64)	0.1111 (ns)	0.5000
	Gastrointestinal infections^1^	8	38	1.71 (0.69–4.19)	0.3107 (ns)	0.7508
	Pneumonias	15	132	0.57 (0.25–1.29)	0.1922 (ns)	0.6192
	Shingles	2	27	0.49 (0.11–2.19)	0.5440 (ns)	0.7769

AIHA, autoimmune hemolytic anemia; CI, confidence interval; CID, combined immunodeficiency; CVID, common variable immunodeficiency; ILD, interstitial lung disease; ITP, immune thrombocytopenic purpura; ns, not significant; OR, odds ratio; PID, primary immunodeficiency disorder; RR, risk ratio; SPAD, specific antibody deficiency.

^+^p < 0.05; **p < 0.01.

^1^Other than with H. pylori.

**Table 4:** Association of patients’ treatment with cancer.

Variable	At least one cancer (*N* = 27)^1^	No cancer (*N* = 192)	OR (95% CI)	p
Immunoglobulin replacement	23	180	0.83 (0.18–3.95)	0.6852 (ns)
Immunosuppressive treatment	8	66	0.8 (0.33–1.93)	0.67 (ns)
-Systemic glucocorticoidmonotherapy	3	27	0.76 (0.22–2.71)	1 (ns)
-csDMARD based regimen	2	27	0.49 (0.11–2.19)	0.544 (ns)
–AZA	1	9	0.78 (0.1–6.43)	1 (ns)
–MTX	1	10	0.7 (0.09–5.67)	1 (ns)
-bDMARD-based regimen	2	11	1.66 (0.35–1.66)	0.6284 (ns)
–RTX	2	6	2.48 (0.47–12.97)	0.2573 (ns)
–TNFi	0	3	0.98 (0.05–19.59)	1 (ns)

AZA, azathioprine; bDMARD, biological disease-modifying antirheumatic drug; csDMARD, conventional synthetic disease-modifying antirheumatic drug; MTX, methotrexate; OR, odds ratio; RTX, rituximab; TNFi, tumor necrosis factor inhibitor.

^1^For patients with cancer, we considered treatment prior to first diagnosis of cancer.

### The Role of Immunological and Genetic Parameters

**Figure 1:**
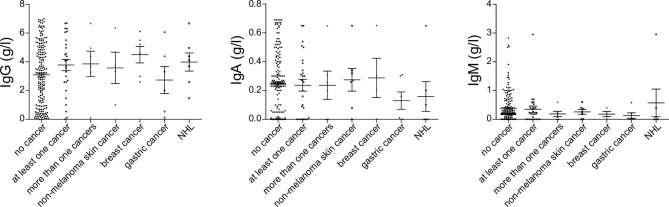
Serum immunoglobulin levels at diagnosis of common variable immunodeficiency (CVID), prior to the introduction of immunoglobulin replacement, in patients with no cancer (*N* = 192), patients with at least one cancer (*N* = 27), more than one type of cancer (*N* = 6), non-melanoma skin cancer (*N* = 7), breast cancer (*N* = 7), gastric cancer (*N* = 6), and non-Hodgkin lymphoma (NHL, *N* = 6). No significant differences could be detected.

**Figure 2:**
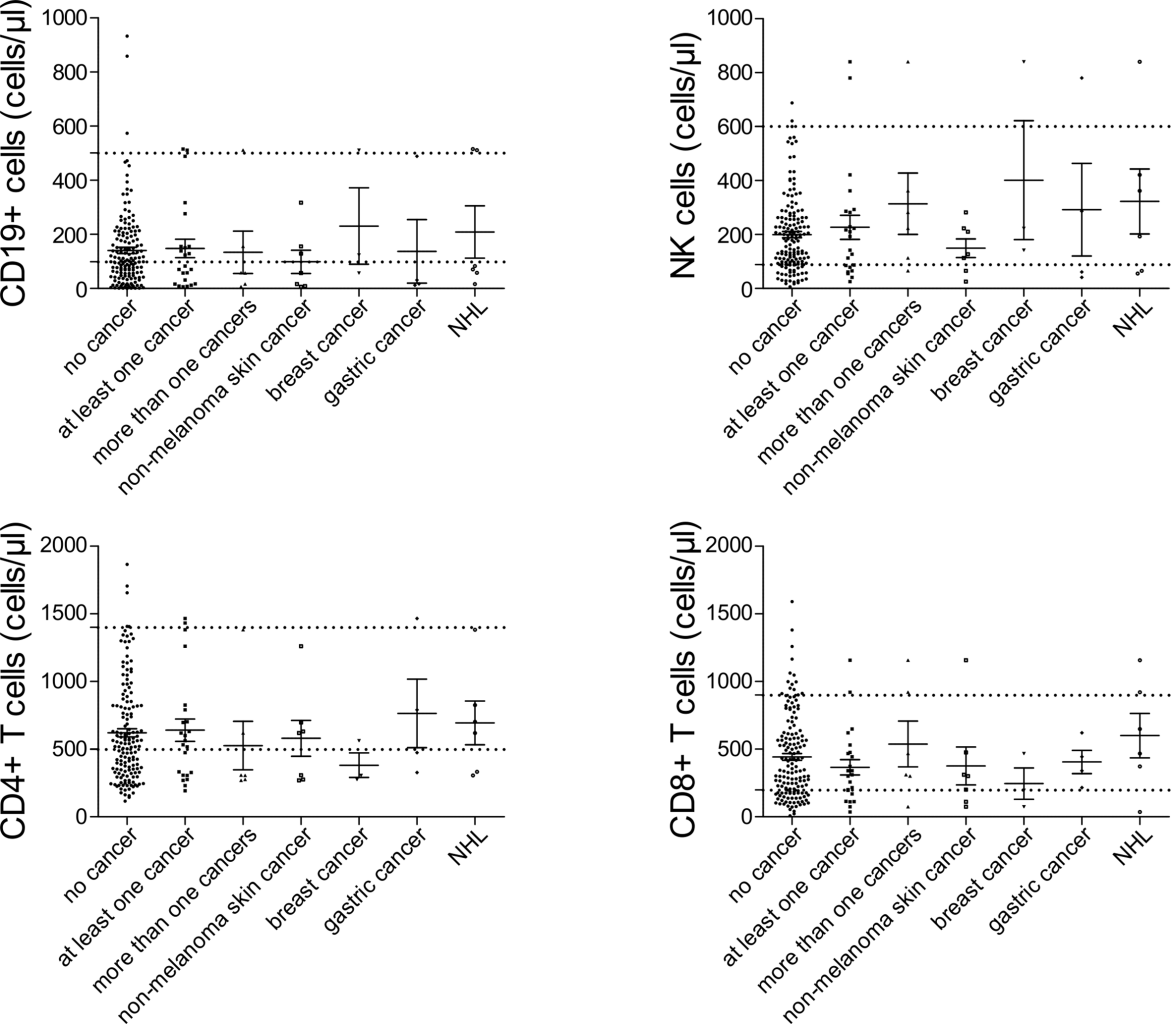
Peripheral lymphocyte subset counts at diagnosis of common variable immunodeficiency (CVID) in patients with no cancer (*N* = 192), patients with at least one cancer (*N* = 27), more than one type of cancer (*N* = 6), non-melanoma skin cancer (*N* = 7), breast cancer (*N* = 7), gastric cancer (*N* = 6), and non-Hodgkin lymphoma (NHL, *N* = 6). No significant differences could be detected.

**Figure 3:**
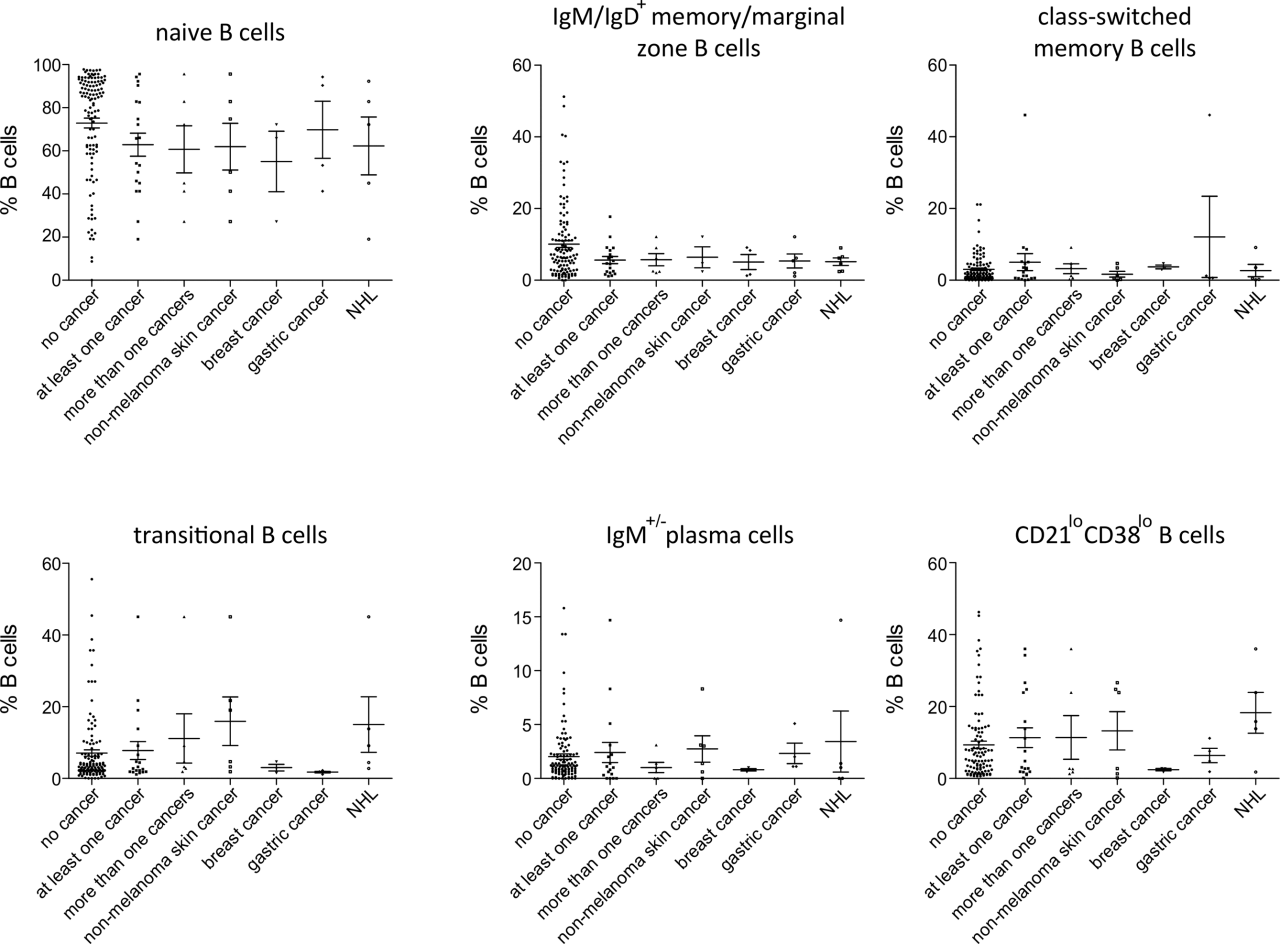
Peripheral B-cell subset in patients with no cancer (*N* = 116), patients with at least one cancer (*N* = 19), more than one type of cancer (*N* = 6), non-melanoma skin cancer (*N* = 6), breast cancer (*N* = 3), gastric cancer (*N* = 4) and non-Hodgkin lymphoma (NHL, *N*=5). No significant differences could be detected.

**Figure 4:**
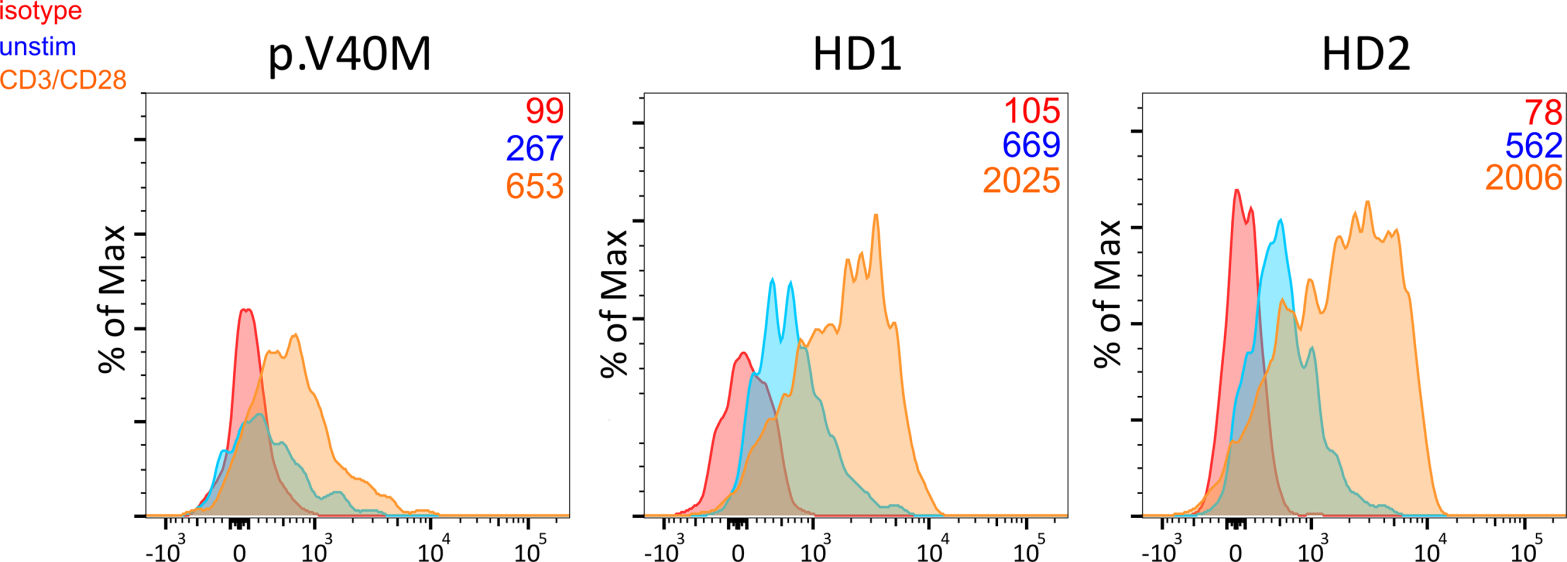
Decreased baseline and CD3/CD28 activation-induced CTLA-4 expression in CD4^+^ CD25^hi^ CD127^lo^ Treg from a patient harboring the c.118G>A (p. V40M) variant in CTLA-4 and two healthy blood donors. Median fluorescence intensity of CTLA-4 is shown (numbers) for each studied subject.

Among patients’ immunological parameters, levels of main classes of immunoglobulins and counts of CD19^+^ B cells, natural killer (NK) cells, and CD4^+^ and CD8^+^ T cells at diagnosis of CVID were available for most patients. We identified no significant differences in the levels of immunoglobulins (**Figure 1**) or the studied lymphocyte or B-cell subsets (**Figures 2**, **3**) between patients with cancer and those without. Furthermore, to identify the genetic background of immunodeficiency, all 27 patients with cancer were initially subjected to genetic testing by means of tNGS. A male patient (patient 3), who harbored a monoallelic variant in *NFKB1* (c.904dupT; p. S302Ffs*7), has been already reported by Schröder et al. (28). Furthermore, tNGS detected a novel *CTLA-4* variant (c.118G>A; p. V40M) in a female with human papillomavirus (HPV)-associated cervical cancer (patient 10). Pathogenicity of this variant was suggested by identifying reduced baseline and activation-induced CTLA-4 expression by patient’s regulatory T cells with flow cytometry (**Figure 4**). Considering allele frequency as well as the combined annotation dependent depletion (CADD) and sorting intolerant from tolerant (SIFT) values of each identified variant, we found 3 predicted pathogenic monoallelic variants in *TNFRSF13B* in two studied patients (patient 1 and patient 25; **Supplementary Table 4**). Variants in *TNFRSF13B*, which encodes the transmembrane activator and calcium-modulating cyclophilin ligand interactor (TACI), are commonly detected in patients with CVID and are considered disease-predisposing rather than disease-causing (29). As the employed tNGS panel included a limited number of CVID and PID-associated genes, we tested 20/27 patients with WES, for whom DNA was available, searching for variants in all known PID-associated genes. In addition to the abovementioned PID-associated variants, WES detected two *TTC7A* variants in a patient (patient 7), who besides cancer displayed recurrent respiratory tract infections and no further CVID-associated manifestations. Biallelic *TTC7A* mutations have been shown to cause combined immunodeficiency associating with early-onset inflammatory bowel disease (IBD) and atresia (30, 31). Despite the fact that the allelic phase of detected *TTC7A* variants has not been tested, lack of any clinical or endoscopic evidence of IBD or structural intestinal defect in this patient makes the diagnosis of TTC7A defect unlikely. All predicted pathogenic variants, identified through tNGS or WES, including the *TNFRSF13B* ones, are listed in **Supplementary Table 4**. Overall, only 2/27 (7.4%) had a definitive genetic diagnosis.

### Cancer Genetic Risk Assessment

To evaluate the possibility of an underlying hereditary cancer predisposition syndrome, we retrospectively evaluated family history documentation, which was available in case of 25/27 studied patients with cancer. In addition, we were able to take family history, focusing on cancer and red flags of hereditary cancer in 20/27, who were not lost to follow-up. Family history data of 25/27 patients with cancer are summarized in **Supplementary Table 5**. Among the studied red flags of hereditary cancer, no patient had a history of consanguinity or an Ashkenazi Jewish ancestry. Elicited family history red flags of hereditary cancer included more often multiple related cancers on the same side of the family or relatively early onset of cancer in patients’ close relatives. Overall, at least one red flag was present in 11/25 (44%) [or 8/20 (40%) in case family history was taken during the study].

Next, we analyzed WES data, which were available for 21/27 CVID patients with cancer, focusing on genes associating with hereditary cancer syndromes (32–34). We detected 18, all monoallelic cancer-associated variants in 12/21 studied subjects, 5 of whom had a family history with at least one hereditary cancer red flag (**Supplementary Table 6**). Most common were missense variants (13/18). With respect to the mechanism of carcinogenesis, most identified variants were identified in genes involved in DNA repair (10/18) and especially double-strand DNA repair (DSR) (6/18; see **Supplementary Table 6**). Interestingly, 3 out of 7 tested patients diagnosed with basal cell carcinoma (patient 1, patient 6, and patient 12) harbored at least one variant in genes involved in DNA repair, which might be relevant for the development of NMSC (35). WES detected no known pathogenic variants, which would lead to the diagnosis of a hereditary cancer syndrome. Genetic variants of unclear significance were however detected in genes such as *CDH1*, *DICER1*, *MEN1*, *RET*, *CREBBP*, *RAD51C*, or *SOS1*, which are linked to autosomal dominant predisposition to cancer (36–43). Given the gene-specific mode of inheritance of cancer susceptibility, the presence of a suggestive family history and the types of diagnosed cancers, 3/21 (14%) WES-tested patients (i.e., patient 1, patient 2, and patient 6; **Supplementary Table 6**) may have a genetic susceptibility to cancer, falling under a known hereditary cancer syndrome. Identified monoallelic variants in genes linked to autosomal recessive predisposition to cancer such as the ones in *RAD50*, *FANCC*, and *FANCM* might also be relevant for cancer risk (44–46).

## Discussion

Previous studies reported a variable prevalence of cancer in patients with CVID and identified lymphomas and gastric cancer as the most common CVID-associated malignancies (9, 10, 47). The relatively high prevalence of those two cancer types in the studied cohort is in line with previously presented case series of CVID. However, the identification of NMSC, and in particular basal cell carcinoma, as the most prevalent CVID-associated cancer deviates from most previous studies (9). This could be explained through the underrecording of cases in previous reports and/or the rising incidence rates of NMSC in the last decades (48). Furthermore, studies on CVID-associated cancers can be confounded by variations between patient cohorts and especially the regional variability in the prevalence of the diverse types of cancer (49, 50). Evaluation of relative occurrence of different forms of cancer in CVID through the calculation of its prevalence can be biased, given the differences in the demographic parameters between studied cohorts and the general population. The estimation of SIRs in the present study highlights the considerably higher occurrence of gastric cancer, NMSC, and NHL in CVID patients as compared to the general population. Besides its association with particular types of cancer, CVID may affect their outcome (9), which needs to be evaluated in studies following up larger numbers of CVID patients with the same forms of cancer.

Despite the fact that the presence of profound T-cell defects precludes the diagnosis of CVID and rather suggests the diagnosis of a combined immunodeficiency, CVID patients can display variable milder cellular defects (18). The degree of cellular immunodeficiency in CVID could associate with the diagnosis of cancer, which would be compatible with the concept of the immune surveillance of tumors (51). In fact, according to a previous report on a cohort of 801 patients with primary hypogammaglobulinemia, including CVID, patients with cancer displayed lower CD8^+^ T-cell counts and patients with non-hematological malignancies had in addition significantly lower B cells (52). In a recent meta-analysis of 48 studies with more than 8,000 CVID patients, those with malignancies tended to display lower percentages of CD8^+^ T cells (3). However, CD8^+^ T-cell percentages were not significantly different, and percentages of the rest of the studied lymphocyte subsets were similar between CVID patients with cancer and those without. Here, evaluation of patients’ immunological parameters at diagnosis of CVID, including B cells, NK cells, and CD8^+^ T-cell counts, revealed no significant differences between patients with cancer and those without. Lack of significant changes in NK or CD8^+^ T cells seemingly contradicts the concept of tumor immune surveillance. Nonetheless, T-cell or NK cell dysfunction has been reported in patients with CVID and may be relevant for CVID-associated carcinogenesis (53–55). Functional characterization of T cells or NK cells is not routinely performed in CVID, and their role as a predictor of carcinogenesis in CVID needs to be evaluated.

The increasing availability of NGS technologies has led to the increasing identification of the genetic background of CVID and to a growing proportion of monogenic disorders, which manifest as CVID. According to recent reports, the proportion of monogenic forms has increased, exceeding 20% of cases (56–58). In the present work, most sequenced patients were sporadic cases. This together with the fact that not all studied patients were subjected to WES may account for the relatively low percentage of patients detected with pathogenic variants. However, in a recent study evaluating the genetic background of immunodeficiency through whole-genome sequencing (WGS) in a large patient cohort mainly consisting of sporadic PID, only approximately 10% of tested patients were identified to harbor pathogenic or likely pathogenic genetic variants (58). Germline mutations in *CTLA4* or *NFKB1* can cause CVID and at the same time associate with an increased risk of malignancy (12, 56). A previous study addressing the genetic cause of CVID in 10 patients with lymphoma revealed a heterogeneous genetic background, including a patient with CTLA4-insufficiency and one with APDS as a consequence of a monoallelic gain-of-function variant in *PIK3CD* (5). Identification of two cancer patients with monogenic CVID in this study, one with CTLA-4 insufficiency and another with NF-κB1 defect, comes in line with previous reports, suggesting the higher risk of malignancy in those monogenic disorders. Similar to the study by Kralickova et al. (5), the prevalence of monogenic disorders among patients with CVID and cancer was relatively low. Despite that, genetic diagnosis of particular disorders, which associate with a higher cancer risk, such as NF-κB1 defect or CTLA-4 insufficiency, should urge treating physicians to consider regular cancer screening.

Besides the identification of the genetic basis of PID and the diagnosis of monogenic inborn errors of immunity, NGS technologies, and in particular higher throughput ones (i.e., WES or WGS), can be useful in identifying cancer risk conferring genetic variants in CVID. The latter may aid in identifying CVID patients at higher risk for malignancies and might consequently lead to intensified cancer screening for this patient subgroup. In the present study, a third of tested patients harbored a predicted pathogenic variant that may lead to hereditary susceptibility to cancer, and less than 25% had in addition a family history, which was suggestive of a hereditary cancer syndrome. With respect to the identified variants, the majority have been identified in genes involved in DNA repair, especially DSR. The latter is in accordance with the previously reported high frequency of genetic variation in DNA repair genes in CVID (57, 58). A genetic background affecting DNA repair would be in line with studies demonstrating chromosomal radiosensitivity (59, 60) and DSR defects in CVID (58) as well as with the identified higher prevalence of NMSC, whose key risk factor is the ultraviolet radiation through its DNA damage-inducing potential (61).

Our study has several limitations. For the calculation of SIR, despite gender and age stratification, the total German population was employed as reference population. Detailed geographical matching of the patients was not possible due to the lack of data from different regions of Germany. However, lack of substantial differences in the incidence of CVID-associated malignancies across different German federal states suggests that geographical matching would have no major influence on SIR values for most forms of cancer. The limited number of patients diagnosed with malignancies and relatively common cancer forms such gastric cancer and lymphomas hampers the identification of immunological risk conferring factors as well as the evaluation of the outcome of particular forms of cancer in CVID. Finally, evaluation of pathogenicity of variations in cancer-associated genes was hampered by the lack of functional testing to identify their likely role in carcinogenesis and sequencing data from family members, which would enable segregation analysis.

Despite the identification of cancer as a main non-infectious manifestation of CVID (9), there is no consensus guideline for cancer screening. Cancer screening could be especially relevant for the outcome of gastric, colorectal, and breast cancer (62), which are common forms of cancer in CVID. In the present study, manifestations of immune dysregulation associated with the diagnosis of cancer in CVID. Furthermore, basal cell carcinoma, which in most cases can be cured with surgical excision (26), associated with the diagnosis of an alternative type of cancer in CVID. Independently of the diagnosis of CVID or an alternative PID, germline mutations have been shown to play an important role in case of early-onset and/or recurrent basal cell carcinomas and to associate with increased risk for other forms of cancer (63, 64). The identification of such cancer-associated manifestations together with the integration of tools/questionnaires (65) and/or relevant information from high-throughput genetic data (i.e., WES or WGS), which are becoming all the more available in daily clinical practice of PID, could aid the development of cost-effective screening programs, which may improve patients’ outcome and reduce cancer-associated mortality in CVID.

## Data Availability Statement

Publicly available datasets were analyzed in this study. These data can be found here: BioProject PRJNA750325 (https://www.ncbi.nlm.nih.gov/bioproject/PRJNA750325).

## Ethics Statement

The studies involving human participants were reviewed and approved by Hannover Medical School. The patients/participants provided their written informed consent to participate in this study (ethics vote number: 5582).

## Author Contributions

Research design: GS and FA; sample collection GS, GA, SH and VP; tNGS data analysis: GS and FA; WES analysis: GS, SvH and GSch; functional assay for CTLA-4 insufficiency, IRA; data analysis: LB, VP, ES, GS and FA; funding acquisition: GS, TW, RS, SvH; writing and contributing to writing of the manuscript GS, FA and all authors. All authors contributed to the article and approved the submitted version.

## Funding

This study was supported by the Germany’s Excellence Strategy (CIBSS–EXC-2189–Project ID 390939984), the “Netzwerke Seltener Erkrankungen” of the German Ministry of Education and Research (BMBF), grant code: GAIN_ 01GM1910A, and the Rosemarie-Germscheid Foundation.

## Conflict of Interest

The authors declare that the research was conducted in the absence of any commercial or financial relationships that could be construed as a potential conflict of interest.

## Publisher’s Note

All claims expressed in this article are solely those of the authors and do not necessarily represent those of their affiliated organizations, or those of the publisher, the editors and the reviewers. Any product that may be evaluated in this article, or claim that may be made by its manufacturer, is not guaranteed or endorsed by the publisher.

## Abbreviations

CTLA-4, cytotoxic T lymphocyte-associated protein 4; CVID, common variable immunodeficiency; DSR, double-strand repair; ILD, interstitial lung disease; NF-κB1, nuclear factor kappa B subunit 1; NHL, non-Hodgkin lymphoma; NMSC, non-melanoma skin cancer; SIR, standardized incidence ratio; tNGS, targeted next-generation sequencing.
