# Supplementary material for: Common Variable Immunodeficiency-Associated Cancers: The Role of Clinical Phenotypes, Immunological and Genetic Factors
Source: Front Immunol. 2022 Feb 17;13:742530. doi: 10.3389/fimmu.2022.742530 (PMC8893227; doi:10.3389/fimmu.2022.742530)
Supplement: Supplementary file 1 [file DataSheet_1.docx]

**Suppl. Table 1**: PID- and cancer-associated genes evaluated with WES

| PID-associated genes: |
| --- |
| *ACD, ACP5, ACTB, ADA, ADA2, ADAM17, ADAR, AICDA, AIRE, AK2, AKT3, ALPI, AP1S3, AP3B1, AP3D1, APCS, APOL1, ARHGEF1, ARPC1B, ATG4A, ATM, ATP6AP1, B2M, BACH2, BCL10, BCL11B, BCL2L1, BCL6, BLM, BLNK, BRCA1, BRCA2, BRIP1, BTK, C1QA, C1QB, C1QC, C1R, C1S, C2, C3, C4A, C4B, C5, C6, C7, C8A, C8B, C8G, C9, CARD11, CARD14, CARD9, CARMIL2, CASP10, CASP8, CCBE1, CCL5, CCR6, CD19, CD247, CD27, CD274, CD28, CD3D, CD3E, CD3G, CD40, CD40LG, CD46, CD55, CD59, CD70, CD79A, CD79B, CD80, CD81, CD86, CD8A, CDC42, CDCA7, CDX1, CEBPE, CFB, CFD, CFH, CFHR1, CFHR2, CFHR3, CFHR4, CFHR5, CFI, CFP, CFTR, CHD7, CIB1, CIITA, CLCN7, CLEC16A, CLPB, COPA, CORO1A, CORO1B, CR2, CSF2RA, CSF2RB, CSF3R, CTC1, CTLA4, CTNNBL1, CTPS1, CTSC, CXCL12, CXCR4, CXCR5, CYBA, CYBB, CYBC1, DBR1, DCLRE1A, DCLRE1B, DCLRE1C, DDX58, DEF6, DGKZ, DKC1, DNAJC21, DNASE1L3, DNASE2, DNMT3B, DOCK11, DOCK2, DOCK8, DUSP2, EFL1, ELANE, EP300, EPCAM, EPG5, ERCC4, ERCC6L2, EXTL3, FAAP24, FADD, FANCA, FANCB, FANCC, FANCD2, FANCE, FANCF, FANCI, FANCL, FANCM, FAS, FASLG, FAT4, FCGR3A, FCGRT, FCHO1, FCN3, FERMT1, FERMT3, FNIP1, FOXN1, FOXP3, FPR1, G6PC3, G6PD, GATA2, GFI1, GINS1, GRAP, GRB2, HAVCR2, HAX1, HDAC4, HELLS, HMOX1, HSPA5, HYOU1, ICOS, ICOSLG, IFIH1, IFNAR1, IFNAR2, IFNG, IFNGR1, IFNGR2, IGHM, IGKC, IGLL1, IKBKB, IKBKE, IKBKG, IKZF1, IL10, IL10RA, IL10RB, IL12B, IL12RB1, IL12RB2, IL17F, IL17RA, IL17RC, IL1RN, IL21, IL21R, IL23R, IL2RA, IL2RB, IL2RG, IL36RN, IL4I1, IL6R, IL6ST, IL7R, INO80, IRAK1, IRAK4, IRF2BP2, IRF3, IRF4, IRF7, IRF8, IRF9, ISG15, ITCH, ITGB2, ITK, JAGN1, JAK1, JAK3, KCNC4, KCNN4, KDM6A, KIDINS220, KMT2A, KMT2D, LAMTOR2, LAT, LCK, LCP2, LIG1, LIG4, LPIN2, LRBA, LRRC32, LRRK2, LSM11, LYST, MAD2L2, MAGT1, MALT1, MAP1LC3B2, MAP3K14, MAPK8, MASP2, MCM10, MCM4, MEFV, MLH1, MOGS, MRTFA, MS4A1, MSH2, MSH5, MSH6, MSN, MTA3, MTHFD1, MVK, MYD88, MYSM1, NBAS, NBN, NCF1, NCF2, NCF4, NCKAP1L, NCSTN, NFAT5, NFE2L2, NFKB1, NFKB2, NFKBIA, NHEJ1, NHP2, NLRC4, NLRP1, NLRP12, NLRP3, NOD2, NOP10, NOS2, NOTCH1, NOTCH2, NSMCE3, OAS1, ORAI1, OSTM1, OTULIN, P2RX7, PALB2, PARN, PAX1, PDCD1, PDCD1LG2, PEPD, PGM3, PIK3AP1, PIK3CD, PIK3CG, PIK3R1, PIK3R4, PLCG2, PLEKHM1, PMS2, PNP, POLA1, POLD1, POLD2, POLE, POLE2, POLR3A, POLR3C, POLR3F, PRDM1, PRF1, PRKCD, PRKD1, PRKDC, PSEN1, PSENEN, PSMB8, PSMG2, PSTPIP1, PTEN, PTPN1, PTPN6, PTPRC, RAB27A, RAC2, RAD50, RAD51, RAD51C, RAG1, RAG2, RANBP2, RASGRP1, RBCK1, REL, RELA, RELB, RFWD3, RFX5, RFXANK, RFXAP, RHOH, RIPK1, RMRP, RNASEH2A, RNASEH2B, RNASEH2C, RNF168, RNU4ATAC, RORA, RORC, RPS6KB2, RPSA, RPTOR, RTEL1, RTP4, SAMD9, SAMD9L, SAMHD1, SBDS, SEC61A1, SEC61A2, SEC61G, SEMA3E, SERPING1, SH2D1A, SH3BP2, SH3KBP1, SKIV2L, SLC29A3, SLC35C1, SLC37A4, SLC39A7, SLC46A1, SLC7A7, SLX4, SMARCAL1, SMARCD2, SNORA31, SNX10, SOCS1, SP110, SPINK5, SPPL2A, SRP54, SRP72, STAT1, STAT2, STAT3, STAT5B, STIM1, STING1, STK11, STK4, STN1, STX11, STXBP2, TAP1, TAP2, TAPBP, TAZ, TBK1, TBX1, TBX21, TCF3, TCIRG1, TCN2, TERC, TERT, TET2, TFRC, TGFB1, TGFB2, TGFB3, TGFBR1, TGFBR2, THBD, TICAM1, TINF2, TIRAP, TLR3, TMC6, TMC8, TNFAIP1, TNFAIP3, TNFRSF10A, TNFRSF11A, TNFRSF13B, TNFRSF13C, TNFRSF17, TNFRSF18, TNFRSF1A, TNFRSF4, TNFRSF9, TNFSF10, TNFSF11, TNFSF12, TNFSF13, TNFSF13B, TOP2B, TP53, TPP1, TPP2, TRAC, TRAF3, TRAF3IP2, TREX1, TRIM22, TRNT1, TTC37, TTC7A, TYK2, UBA1, UNC13D, UNC93B1, UNG, USB1, USP18, USP8, VAV1, VAV2, VPS13B, VPS45, WAS, WDR1, WIPF1, WNT5A, WRAP53, XCL1, XIAP, ZAP70, ZBTB24, ZNF341, ZNFX1* |
| Cancer-associated genes: |
| *ABRAXAS1, AIP, AKT1, ALK, ANKRD26, APC, ATM, BAP1, BARD1, BLM, BMPR1A, BRAF, BRCA1, BRCA2, BRIP1, BUB1B, CDC73, CDH1, CDK4, CDKN1B, CDKN1C, CDKN2A, CEBPA, CHEK2, CREBBP, CTR9, DDB2, DDX41, DICER1, DIS3L2, ETV6, ERCC2, FANCA, FANCB, FANCC, FANCD2, FANCE, FANCF, FANCG, FANCI, FANCL, FANCM, FH, FLCN, GATA2, GPC3, HOXA11, IKZF1, KIT, KRAS, MAP3K1, MAX, MBD4 , MECOM, MEN1, MET, MITF, MLH1, MRE11, MSH2, MSH3, MSH6, MUTYH, NBN, NF1, NF2, NRAS, NTHL1, PALB2, PAX5, PHOX2B, PIK3CA, PMS2, POLD1, POLE, POLH, POLQ, PPM1D, PRKAR1A, PTCH1, PTCH2, PTEN, PTPN11, RAD50, RAD51, RAD51C, RAD51D, RAF1, RB1, RBBP6, RECQL4, REST, RET, RHBDF2, RINT1, RIT1, RUNX1, SAMD9, SAMD9L, SDHAF2, SDHB, SDHC, SDHD, SLX4, SMAD4, SMARCA4, SMARCB1, SOS1, SRP72, STK11, SUFU, TERC, TERT, TMEM127, TP53, TRIP13, TSC1, TSC2, U2AF1, USP9X, VHL, WT1, XPA, XRCC2* |

**Suppl. Table 2**: clinical characteristics of 27 CVID patients with cancer

| Pat. Nr. | Gender | Age at reporting/death^†^ | Age at diagnosis of CVID | CVID manifestations | Age at first diagnosis of 1st cancer | 1^st^ cancer | Age at first diagnosis of 2^nd^ cancer | 2^nd^ cancer | Age at first diagnosis of 3^rd^ cancer | 3^rd^ cancer |
| --- | --- | --- | --- | --- | --- | --- | --- | --- | --- | --- |
| 1 | M | 42 | 37 | recurrent pneumonias, lymphadenopathy | 37 | basal cell carcinoma, treatment: complete surgical excision |  |  |  |  |
| 2 | F | 58 | 37 | recurrent bronchitis/sinusitis, lymphadenopathy, splenomegaly, ITP, GLILD | 52 | multiple basal cell carcinomas, treatment: complete surgical excision, adjuvant RT in one lesion |  |  |  |  |
| 3 | M | 61 | 38 | recurrent bronchitis, pneumonias & gastrointestinal infections, ITP, enteropathy, lymphenopathy | 50 | basal cell carcinoma, treatment: complete surgical excision | 55 | B-NHL (diffuse large B-cell lymphoma; EBV-neg.), treatment: R-CHOP, outcome: CR, RF |  |  |
| 4 | M | 43^†^ | 22 | recurrent bronchitis, pneumonias & gastrointestinal infections, bronchiectasis, enteropathy (Crohn-like), splenomegaly, lymphadenopathy | 42 | B-NHL (high grade B-cell Lymphoma; EBV-pos.), treatment: R-CHOP & RT, outcome: died of sepsis after 1st R-CHOP |  |  |  |  |
| 5 | M | 48 | 31 | recurrent bronchitis & pneumonias, lymphadenopathy, GLILD | 40 | B-NHL (diffuse large B-cell Lymphoma; EBV-neg.), treatment: R-CHOP, outcome: CR, RF |  |  |  |  |
| 6 | F | 58 | 44 | recurrent bronchitis & pneumonias, bronchiectasis, topic dermatitis, allergic rhinitis & asthma | 42 | breast cancer, stage IA (T1N0M0), treatment: lumpectomy, WBRT, adjuvant tamoxifen, outcome: axillary cutaneous and pulmonary metastasis 2 years after diagnosis | 43 | colon cancer stage IIA (T3N0M0), treatment: surgical resection, adjuvant 5FU/LV for 6 months | 50 | multiple basal cell carcinomas, treatment: complete surgical excision |
| 7 | F | 78 | 66 | recurrent bronchitis & pneumonias, bronchiectasis | 59 | breast cancer, stage IA (T1N0M0), treatment: lumpectomy, WBRT adjuvant anastrozole, outcome: RF | 60 | B-NHL (diffuse large B-cell Lymphoma) , treatment: R-CHOP, outcome: CR, RF |  |  |
| 8 | F | 72 | 57 | recurrent bronchitis & pneumonias | 50 | breast cancer, stage IA (T1N0M0), treatment: lumpectomy WBRT, adjuvant anastrozole, outcome: RF |  |  |  |  |
| 9 | F | 82 | 52 | recurrent pneumonias | 56 | breast cancer, stage IA (T1N0M0), treatment: lumpectomy, WBRT, adjuvant anastrazole, outcome: RF |  |  |  |  |
| 10 | F | 32 | 25 | recurrent bronchitis, warts (verruca plantaris), enteropathy, atopic dermatitis, alopecia areata, lymphadenopathy, JIA | 31 | cervical cancer, stage 1A1, HPV16-pos., no LVSI, treatment: fertility sparing approach: radical trachelectomy, outcome: RF |  |  |  |  |
| 11 | F | 48 | 41 | recurrent bronchitis, reactive arthritis, lymphadenopathy | 40 | gastric cancer, stage 1A (pT1bN0M0), proximal, *H. pylori*-pos., treatment: total gastrectomy (R0 resection), outcome: RF |  |  |  |  |
| 12 | M | 69^†^ | 44 | recurrent bronchitis & sinusitis, atrophic gastritis, celiac disease, enteropathy | 68 | basal cell carcinoma, treatment: complete surgical excision | 69 | gastric cancer, stage IB, (pT1N1M0), total gastrectomy (R0, D2 resection), adjuvant FOLFOX, outcome: died 6 months after diagnosis |  |  |
| 13 | M | 37 | 5 | recurrent bronchitis, sinusitis & pneumonias, AIHA | 36 | gastric cancer, stage: IIB (T4aN0M0), treatment: perioperative chemotherapy (FLOT) and subtotal gastrectomy, D2 lymphadenectomy, outcome: metachronous dissemination with peritoneal metastasis progressing despite pembrolizumab-treatment, actual treatment: ramucirumab + paclitaxel |  |  |  |  |
| 14 | M | 55 | 39 | recurrent bronchitis & gastrointestinal infections | 37 | gastric cancer, stage IB (pT2N0M0) G2, treatment: total gastrectomy (R0 resection), outcome: RF |  |  |  |  |
| 15 | M | 38 | 28 | recurrent bronchitis & pneumonias, T1D, atrophic gastritis, autoimmune thyreoiditis | 32 | gastric carcinoid tumor type 1, enterochromaffin-like (ECL) cell origin, stage: 1, (T1N0M0), treatment: endoscopic resection, outcome: RF |  |  |  |  |
| 16 | F | 36 | 12 | recurrent bronchitis, pneumonias & gastrointestinal infections, ITP, AIHA, GLILD, lymphadenopathy, atopic dermatitis | 36 | gastric carcinoid tumor type 1, stage 1 (T1N0M0), treatment: endoscopic resection, outcome: n.a. |  |  |  |  |
| 17 | F | 59 | 54 | recurrent bronchitis & pneumonias, bronchiectasis, lymphadenopathy, splenomegaly, ITP | 42 | multiple basal cell carcinomas, treatment: complete surgical excision |  |  |  |  |
| 18 | F | 55^†^ | 40 | recurrent bronchitis & pneumonias | 53 | rectal adenocarcinoma, stage: IVC, T2N2bM1c, treatment: prophylactic surgical resection, 1^st^ line treatment with CAPOX, outcome: died of chemotherapy-induced alveolitis |  |  |  |  |
| 19 | F | 63 | 54 | recurrent bronchitis, chronic polyarthritis, atopic dermatitis | 60 | rectal adenocarcinoma, stage 0 (TisN0M0), treatment: endoscopic resection | 60 | multiple basal cell carcinomas, treatment: complete surgical excision |  |  |
| 20 | F | 74 | 58 | recurrent bronchitis & pneumonias, bronchiectasis, GLILD, lymphadenopathy | 67 | rectal adenocarcinoma, stage IIA (T3N0M0), G2, treatment: subtotal colectomy, adjuvant chemotherapy with FOLFOX, outcome: 6 years after first diagnosis, pulmonary metastasis and treatment with bevacizumab + capecitabine |  |  |  |  |
| 21 | F | 60 | 32 | recurrent pneumonias, GLILD | 33 | renal cell carcinoma, stage: I, (pT1N0M0), treatment: local surgical tumor excision, outcome: RF | 50 | basal cell carcinoma, treatment: complete surgical excision | 60 | breast cancer, stage IIA (pT2pN0 (0/1)M0), G2, treatment: mastectomy,WBRT, adjuvant tamoxifen and then letrozole, outcome: relapse after more 10 years with lung metastasis: 1^st^ line paclitaxel weekly |
| 22 | F | 71 | 66 | recurrent bronchitis, GLILD, lymphadenopathy | 65 | renal cell carcinoma, stage: I, T1aN0M0, treatment: radical nephrectomy, outcome: RF | 71 | low malignant B-NHL (new diagnosis) |  |  |
| 23 | F | 59^†^ | 56 | recurrent bronchitis & pneumonias | 58 | small cell lung carcinoma, limited disease, treatment: concurrent chemotherapy (cisplatin and etoposide) and RT followed by whole brain radiotherapy.  Relapse < 6 months with liver metastases: 2^nd^ line treatment with Topotecan (iv), progression disease in liver: 3^rd^ line treatment with nivolumab, outcome: died of tumor progression |  |  |  |  |
| 24 | M | 30 | 29 | recurrent bronchitis & pneumonias, psoriasis arthritis | 29 | testicular seminoma, stage I, (pT1a, cN0, M0), treatment: orchiectomy, adjuvant chemotherapy with 1Cy carboplatin, outcome: RF |  |  |  |  |
| 25 | F | 47^†^ | 45 | recurrent bronchitis, pneumonias & gastrointestinal infections, bronchiectasis, AIHA, autoimmune thyreoiditis, atrophic gastritis, leukocytoclastic vasculitis, lymphadenopathy, splenomegaly | 46 | T-NHL, stage: IIB, treatment: CHOP, vorinostat, outcome: died of neutropenic sepsis in the course of chemotherapy. |  |  |  |  |
| 26 | F | 55 | 42 | granulomatous hepatitis | 45 | vulvar cancer, stage IB (pT1bN0M0) without risk factors, treatment: partial vulvectomy, outcome: RF |  |  |  |  |
| 27 | F | 73 | 46 | recurrent bronchitis, rheumatoid arthritis, lymphadenopathy | 69 | vulvar cancer, stage IB (pT1bN0M0) without risk factors, treatment: partial vulvectomy, outcome: RF |  |  |  |  |
| 5-FU/LV, 5-fluorouracil and high-dose leucovorin; AIHA, autoimmune hemolytic anemia; CAPOX, capecitabine, oxaliplatin; CHOP, cyclophosphamide doxorubicin vincristine prednisone; CR, complete remission; Cy: cycle of treatment; EBV, Epstein–Barr virus; F, female; FLOT, docetaxel, oxaliplatin, leucovorin & 5-fluorouracil; FOLFOX, 5-fluorouracil, oxaliplatin; GLILD, granulomatous-lymphocytic interstitial lung disease; HPV, human papillomavirus; ITP, immune thrombocytopenic purpura; LVSI, lymphovascular invasion; M, male; neg., negative; NHL, non-Hodgkin lymphoma; pos., positive; R-CHOP, rituximab- cyclophosphamide doxorubicin vincristine prednisone; RF, relapse-free; RT, radiotherapy; T1D, type 1 diabetes; WBRT, whole breast radiotherapy | | | | | | | | | | |

**Suppl. Table 3:** Mean age at diagnosis and prevalence of CVID associated cancers as compared to data from the 570 reference population (24)

|  |  | gastric cancer | | NMSC | | NHL | |
| --- | --- | --- | --- | --- | --- | --- | --- |
|  | gender | female | male | female | male | female | male |
| reference population | median age (years) | 76 | 72 | 73 | 75 | 72 | 70 |
|  | prevalence (%) | 0.03 | 0.05 | 1.17 | 1.31 | 0.07 | 0.09 |
| CVID-cohort (*N*=219) | median age (years) | 38 | 34.5 | 50 | 50 | 60 | 42 |
|  | prevalence (%) | 1.56 | 4.40 | 3.91 | 3.30 | 2.34 | 3.30 |

**Suppl. Table 4**: predicted pathogenic variants in PID-related genes, identified in 27 patients with CVID and cancer. All except for the TTCA7 variants were initially detected with tNGS (see Sogkas et al, reference 19)

| **Pat.**  **ID** | **gene** | **transcript ID** | **transcript variant** | **protein variant** | **dbSNP-ID** | **gnomAD frequency** | **CADD**  **Score** | **PolyPhen2**  **Pred** | **SIFT**  **Pred** |
| --- | --- | --- | --- | --- | --- | --- | --- | --- | --- |
| 1 | *TNFRSF13B* | NM_012452.3 | c.204dup | p.(Leu69Thrfs*12) | [rs72553875](https://www.ncbi.nlm.nih.gov/snp/rs72553875) | 0.0003090 | 22.8 | probably damaging | D |
| 1 | *TNFRSF13B* | NM_012452.3 | c.1306C>G | p.(Cys104Arg) | rs34557412 | 0.003922 | 25.8 | probably damaging | D |
| 3 | *NFKB1* | NM_003998.3 | c.904dup | p.(Ser302Phefs*7) | [rs773694113](https://www.ncbi.nlm.nih.gov/projects/SNP/snp_ref.cgi?rs=rs773694113) | 0.000006579 | 32 | probably damaging | D |
| 7 | *TTC7A* | NM_020458.3 | c.1817A>G | p.(Lys606Arg) | [rs139010200](https://www.ncbi.nlm.nih.gov/projects/SNP/snp_ref.cgi?rs=rs139010200) | 0.002083 | 29.5 | probably damaging | D |
| 7 | *TTC7A* | NM_020458.3 | c.2014T>C | p.(Ser672Pro) | [rs149602485](https://www.ncbi.nlm.nih.gov/projects/SNP/snp_ref.cgi?rs=rs149602485) | 0.002090 | 24.9 | possibly damaging | D |
| 10 | *CTLA4* | NM_001037631.2 | c.118G>A | p.(Val40Met) | . | . | 24.2 | possibly damaging | D |
| 25 | *TNFRSF13B* | NM_012452.ex.4 | c.542C>A | p. (Ala181Glu) | rs72553883 | 3,70E-03 | 22.8 | benign | D |
| 26 | *RELA* | NM_001145138.2 | c.622G>A | p. (Glu208Lys) | NA | NA | 34 | probably damaging | D |
| 26 | *GATA2* | NM_001145662.1 | c.1343C>A | p. (Ser448Tyr) | NA | NA | 25 | possibly damaging | D |
| CADD, combined annotation dependent depletion; D, damaging; gnomAD, Genome Aggregation Database; NA, not applicable; Pat. ID, patient identification number; pred., prediction; ref.seq., reference sequence; SIFT, sorting intolerance from tolerance | | | | | | | | | |

**Suppl. Table 5**: Family history of cancer, including red flags of hereditary cancer of 25 CVID patients with cancer

| Pat. ID | WES available  (1= Yes) | Family  History | First-degree relative | | | | | Mother side | | | | | | Father side | | | | | | Multiple | Young < 50 years | Rare | red flag |
| --- | --- | --- | --- | --- | --- | --- | --- | --- | --- | --- | --- | --- | --- | --- | --- | --- | --- | --- | --- | --- | --- | --- | --- |
|  |  |  | Breast cancer | Uterine cancer | Colon/Rectal cancer | Colorectal Polyps | Other cancer | Breast cancer | Ovarian cancer | Uterine cancer | Colon/Rectal cancer | Colorectal Polyps | Other cancer | Breast cancer | Ovarian cancer | Uterine cancer | Colon/Rectal cancer | Colorectal Polyps | Other cancer | 2 or more breast cancers | Breast/Colorectal/endometrial | Ovarian/>10 Polyps |  |
| **1** | 1 | **during** | 0 | 0 | 0 | 0 | **1 (father died gastric cancer at the age of 49 years)** | 0 | 0 | 0 | 0 | 0 | 0 | 0 | 0 | 0 | 0 | 0 | 0 | 0 | 1 | 0 | 1 |
| **2** | 1 | **during** | 0 | 0 | 0 | 0 | 0 | 0 | 0 | 0 | **1 (grandmother died of colorectal cancer at the age of 89 )** | 0 | 0 | 0 | 0 | 0 | 0 | 0 | 0 | 0 | 0 | 0 | 0 |
| **3** | 1 | **during** | 0 | **1 (mother with endometrial cancer diagnosed at the age of approximately 85 years)** | 0 | 0 | 0 | 0 | 0 | 0 | 0 | 0 | 0 | 0 | 0 | 0 | 0 | 0 | 0 | 0 | 0 | 0 | 0 |
| **4** | 1 | **retrosp** | **1 (mother diagnosed with breast cancer at the age of 36)** | 0 | 0 | 0 | 0 | **1 (grandmother died of breast cancer, age?)** | 0 | 0 | 0 | 0 | 0 | 0 | 0 | 0 | 0 | 0 | 0 | 1 | 1 | 0 | 1 |
| **5** | 1 | **during** | 0 | 0 | 0 | 0 | 0 | 0 | 0 | 0 | 0 | 0 | 0 | 0 | 0 | 0 | 0 | 0 | 0 | 0 | 0 | 0 | 0 |
| **6** | 1 | **during** | 0 | 0 | 0 | 0 | **1 (father died gastric cancer at the age of 60 years)** | 0 | 0 | 0 | 0 | 0 | 0 | **1 (aunt died of breast cancer at the age of 50)** | 0 | 0 | 0 | 0 | **1 (grandfather with gastric cancer)** | 1 | 1 | 0 | 1 |
| **7** | 0 | **during** | 0 | 0 | 0 | 0 | 0 | 0 | 0 | 0 | 0 | 0 | 0 | 0 | 0 | 0 | 0 | 0 | 0 | 0 | 0 | 0 | 0 |
| **8** | 1 | **during** | 0 | 0 | **1 (mother died of colorectal cancer at the age of 68, diagnosed 65)** | 0 | 0 | 0 | 0 | 0 | **1 (aunt-mother's sister died of colorectal cancer at the age of approximately 75 years)** | 0 | **1 (grandmother died of gastric cancer at the age of 48 years)** | 0 | 0 | 0 | 0 | 0 | **1 (grandmother died of melanoma, old>80 years)** | 1 | 1 | 0 | 1 |
| **9** | 1 | **during** | 0 | 0 | 0 | 0 | 0 | 0 | 0 | 0 | 0 | 0 | 0 | 0 | 0 | 0 | 0 | 0 | 0 | 0 | 0 | 0 | 0 |
| **10** | 1 | **during** | 0 | 0 | 0 | **1 (father with a history of colonic polypectomy at the of 70 years, currently 78 years old)** | 0 | 0 | 0 | 0 | 0 | 0 | **1 (grandmother died of ovarian cancer at the age of 74 years)** | 0 | 0 | 0 | 0 | 0 | **1 (half-sister with lymphoma diagnosed at the age of 52 years)** | 1 | 0 | 1 | 1 |
| **11** | 0 | **during** | 0 | 0 | 0 | 0 | **1 (brother diagnosed with lymphoma (NHL) at the age of 50 years)** | 0 | 0 | 0 | 0 | 0 | 0 | 0 | 0 | 0 | 0 | 0 | 0 | 0 | 1 | 0 | 1 |
| **12** | 1 | **retrosp** | **1 (mother diagnosed with breast cancer at the age of 50)** | 0 | 0 | 0 | 0 | 0 | 0 | 0 | 0 | 0 | 0 | 0 | 0 | 0 | 0 | 0 | 0 | 1 | 1 | 0 | 1 |
| **13** | 0 | **during** | 0 | 0 | **1 (father with a history of colorectal cancer at diagnosed at the age of 47 years)** | 0 | 0 | 1 | 0 | 0 | 0 | 0 | 0 | 0 | 0 | 0 | **1 (grandfather died of colorectal cancer)** | 0 | 0 | 1 | 1 | 0 | 1 |
| **14** | 1 | **during** | 0 | 0 | 0 | 0 | 0 | 0 | 0 | 0 | 0 | 0 | 0 | 0 | 0 | 0 | 0 | 0 | 0 | 0 | 0 | 0 | 0 |
| **15** | 1 | **during** | 0 | 0 | 0 | 0 | 0 | 0 | 0 | 0 | 0 | 0 | 0 | 0 | 0 | 0 | 0 | 0 | 0 | 0 | 0 | 0 | 0 |
| **16** | 1 | n.a. | n.a. | n.a. | n.a. | n.a. | n.a. | n.a. | n.a. | n.a. | n.a. | n.a. | n.a. | n.a. | n.a. | n.a. | n.a. | n.a. | n.a. |  |  |  |  |
| **17** | 1 | **during** | 0 | 0 | 0 | 0 | 0 | 0 | 0 | 0 | 0 | 0 | **1 (grandmother died of hepatoma at the age of approximately 80 years)** | 0 | 0 | 0 | 0 | 0 | 0 | 0 | 0 | 0 | 0 |
| **18** | 0 | n.a. | n.a. | n.a. | n.a. | n.a. | n.a. | n.a. | n.a. | n.a. | n.a. | n.a. | n.a. | n.a. | n.a. | n.a. | n.a. | n.a. | n.a. |  |  |  |  |
| **19** | 1 | **during** | 0 | 0 | **1 (father with colon cancer, age?)/1 (mother died of colorectal cancer at the age of 67 years)** | 0 | **1 (father with prostate cancer, age?)** | 0 | 0 | 0 | 0 | 0 | 0 | 0 | 0 | 0 | **1 (grandfather with colorectal carcinoma)** | 0 | 0 | 1 | 0 | 0 | 1 |
| **20** | 1 | **retrosp** | 0 | 0 | 0 | 0 | 0 | 0 | 0 | 0 | 0 | 0 | 0 | 0 | 0 | 0 | 0 | 0 | 0 | 0 | 0 | 0 |  |
| **21** | 0 | **during** | 0 | 0 | 0 | 0 | 0 | 0 | 0 | 0 | 0 | 0 | 0 | 0 | 0 | 0 | 0 | 0 | 0 | 0 | 0 | 1 | 1 |
| **22** | 1 | **during** | 0 | 0 | 0 | 0 | **1 (brother with esophageal cancer)** | 0 | 0 | 0 | 0 | 0 | 0 | 0 | 0 | 0 | 0 | 0 | 0 | 0 | 0 | 0 | 0 |
| **23** | 0 | **retrosp** | 0 | 0 | 0 | 0 | 0 | 0 | 0 | 0 | 0 | 0 | 0 | 0 | 0 | 0 | 0 | 0 | 0 | 0 | 0 | 0 | 0 |
| **24** | 1 | **during** | **1 (mother diagnosed with breast cancer ath the age of 45 years)** | 0 | 0 | 0 | 0 | 0 | 0 | 0 | 0 | 0 | 0 | 0 | 0 | 0 | 0 | 0 | 0 | 1 | 1 | 0 | 1 |
| **25** | 1 | **retrosp** | 0 | 0 | 0 | 0 | 0 | 0 | 0 | 0 | 0 | 0 | 0 | 0 | 0 | 0 | 0 | 0 | **1 (grandfather died of cancer with unknown primary)** | 0 | 0 | 0 | 0 |
| **26** | 1 | **during** | 0 | 0 | 0 | 0 | 0 | 0 | 0 | 0 | 0 | 0 | 0 | 0 | 0 | 0 | 0 | 0 | 0 | 0 | 0 | 0 | 0 |
| **27** | 1 | **during** | 0 | 0 | 0 | 0 | **1 (father died of lung cancer at the age of 68)** | 0 | 0 | 0 | 0 | 0 | 0 | 0 | 0 | 0 | 0 | 0 | 0 | 0 | 0 | 0 | 0 |

**Suppl. Table 6**: Predicted pathogenic variants in cancer-associated genes identified with WES in 21 CVID patients with cancer

| **Pat.**  **ID** | **family history suggestive of hereditary cancer** | **gene** | **protein function/ hereditary cancer syndrome** | **gene**  **references** | **transcript ID** | **transcript variant** | **protein variant** | **dbSNP-ID** | **gnomAD frequency** | **CADD**  **Score** | **PolyPhen2**  **Pred.** | **SIFT**  **Pred.** |
| --- | --- | --- | --- | --- | --- | --- | --- | --- | --- | --- | --- | --- |
| 1 | Y | *CDH1* | cell cycle control- G1/S and G2/M phase transition; DNA repair – DNA damage response, DSB repair (AD) | S1, S2 | NM_004360.5 | c.2635G>A | p.(Gly879Ser) | [rs200911775](https://www.ncbi.nlm.nih.gov/projects/SNP/snp_ref.cgi?rs=rs200911775) | 0.0001249 | 28.2 | probably damaging | D |
| 4 | Y | *TSC1* | regulation of the mTOR pathway, which is involved in cell proliferation (AD) | S3 | NM_001162427.1 | c.2473-3_2473-2insTA | p.? | n.a. | n.a. | n.a. | n.a. | n.a. |
| 5 | N | *RAD50* | DSB repair, NHEJ/ RAD50 deficiency (AR) | S4-S6 | NM_005732.4 | c.1336A>G | p.(Lys446Glu) | [rs149217423](https://www.ncbi.nlm.nih.gov/projects/SNP/snp_ref.cgi?rs=rs149217423) | 0.0002299 | 27.4 | probably damaging | D |
|  |  | *FANCM* | ATR-mediated DNA damage checkpoint, Fanconi anemia (AR) | S7 | NM_020937.3 | c.171G>C | p.(Leu57Phe) | [rs142007602](https://www.ncbi.nlm.nih.gov/projects/SNP/snp_ref.cgi?rs=rs142007602) | 0.001551 | 21.4 | benign | T |
| 6 | Y | *BLM* | DSB repair, homologous recombination repair/ Bloom syndrome (AR) | S4, S8 | LRG_20t1 | c.98+1G>C | p.? | [rs750293380](https://www.ncbi.nlm.nih.gov/projects/SNP/snp_ref.cgi?rs=rs750293380) | 0.00002671 | 24.8 | n.a. | n.a. |
|  |  | *FANCC* | DSB repair, homologous recombination, Fanconi anemia (AR) | S9 | NM_000136.3 | c.632C>G | p.(Pro211Arg) | rs140781259 | 0.001361 | 23 | possibly damaging | D |
|  |  | *RAD51D* | DSB repair, homologous recombination repair (AD) | S10, S11 | ENST00000335858 | c.362A>G | p.(Glu121Gly) | rs28363284 | 0.009636 | 24 | n.a. | n.a. |
| 10 | N | *DIS3L2* | RNA metabolism/cell growth and proliferation/Perlman syndrome (AR) | S12 | NM_001257281.1 | c.1447C>G | p.(Arg483Gly) | [rs186865544](https://www.ncbi.nlm.nih.gov/projects/SNP/snp_ref.cgi?rs=rs186865544) | 0.0008412 | 26.8 | probably damaging | D |
| 12 | Y | *FANCM* | ATR-mediated DNA damage checkpoint, Fanconi anemia (AR) | S7 | NM_020937.3 | c.5195_5196insC | p.(Lys1732Asnfs*23) | n.a. | n.a. | n.a. | n.a. | n.a. |
| 14 | N | *RAD50* | DSB repair, NHEJ, RAD50 deficiency (AR) | S4-S6 | NM_005732.4 | c.2840T>C | p.(Ile947Thr) | [rs150401251](https://www.ncbi.nlm.nih.gov/projects/SNP/snp_ref.cgi?rs=rs150401251) | 0.00007885 | 22.5 | benign | T |
| 17 | N | *RET* | cell differentiation, growth, survival, MEN2 (AD) | S13 | NM_020975 | c.2641C>G | p.(Leu881Val) | n.a. | n.a. | 23.2 | probably damaging | D |
| 20 | N | *FANCM* | ATR-mediated DNA damage checkpoint, Fanconi anemia (AR) | S7 | NM_020937.3 | c.869T>C | p.(Ile290Thr) | [rs377303950](https://www.ncbi.nlm.nih.gov/projects/SNP/snp_ref.cgi?rs=rs377303950) | 0.00007227 | 22.9 | benign | D |
| 24 | Y | *MRE11* | DSB repair, homologous recombination repair (AR) | S14 | NM_005591.3 | c.529G>A | p.(Ala177Thr) | [rs142996063](https://www.ncbi.nlm.nih.gov/snp/rs142996063) | 0.0001117 | 26.3 | probably damaging | D |
|  |  | *CREBBP* | histone acetyltransferase, regulation of gene expression/ Rubinstein-Taybi syndrome (AD) | S15, S16 | NM_004380.3 | c.1406_1408del | p.(Ser469del) | n.a. | n.a. |  | n.a. | n.a. |
|  |  | *DICER1* | regulation of the expression of tumor suppressor genes and oncogens/ DICER1 syndrome (AD) | S17 | NM_177438.2 | c.4699C>G | p.(Leu1567Val) | n.a. | n.a. | 25.5 | probably damaging | D |
| 25 | N | *SOS1* | KRAS regulation/ Noonan syndrome (AD) | S18, S19 | NM_005633.3 | c.34A>T | p.(Ser12Cys) | [rs751776207](https://www.ncbi.nlm.nih.gov/projects/SNP/snp_ref.cgi?rs=rs751776207) | 0.000006578 | 26.3 | probably damaging | D |
|  |  | *MEN1* | tumor suppressor/ MEN1 (AD) | S20 | NM_130800.2 | c.1366-4C>T | p.? | n.a. | n.a. | n.a. | n.a. | n.a. |
| 27 | N | *ERCC2* | SS repair- nucleotide excision DNA repair/ Trichothiodystrophy (AR) | S21 | NM_000400.4 | c.1541T>C | p.(Ile514Thr) | [rs762741848](https://www.ncbi.nlm.nih.gov/projects/SNP/snp_ref.cgi?rs=rs762741848) | 0.000006576 | 22,5 | benign | T |
| AD, autosomal dominant; AR, autosomal recessive; CADD, combined annotation dependent depletion; D, damaging; gnomAD, Genome Aggregation Database; N, absent; NA, not applicable; Pat. ID, patient identification number; pred., prediction; ref.seq., reference sequence; SIFT, sorting intolerance from tolerance; Y, present | | | | | | | | | | | | |

**Suppl. Table 6: References**

S1. Hansford S, Kaurah P, Li-Chang H, Woo M, Senz J, Pinheiro H, et al. Hereditary Diffuse Gastric Cancer Syndrome: CDH1 Mutations and Beyond. JAMA Oncol. 2015;1(1):23-32. doi: 10.1001/jamaoncol.2014.168.

S2. Li J, Xu X. DNA double-strand break repair: a tale of pathway choices. Acta Biochim Biophys Sin (Shanghai). 2016;48(7):641-6. doi: 10.1093/abbs/gmw045.

S3. Rosset C, Netto CBO, Ashton-Prolla P. TSC1 and TSC2 gene mutations and their implications for treatment in Tuberous Sclerosis Complex: a review. Genet Mol Biol. 2017;40(1):69-79. doi: 10.1590/1678-4685-GMB-2015-0321.

S4. Risinger MA, Groden J. Crosslinks and crosstalk: human cancer syndromes and DNA repair defects. Cancer Cell. 2004;6(6):539-45. doi: 10.1016/j.ccr.2004.12.001.

S5. Ragamin A, Yigit G, Bousset K, Beleggia F, Verheijen FW, de Wit MY, et al. Human RAD50 deficiency: Confirmation of a distinctive phenotype. Am J Med Genet A. 2020;182(6):1378-1386. doi: 10.1002/ajmg.a.61570.

S6. Heikkinen K, Rapakko K, Karppinen SM, Erkko H, Knuutila S, Lundán T, et al. RAD50 and NBS1 are breast cancer susceptibility genes associated with genomic instability. Carcinogenesis. 2006;27(8):1593-9. doi: 10.1093/carcin/bgi360.

S7. Bogliolo M, Bluteau D, Lespinasse J, Pujol R, Vasquez N, d'Enghien CD, et al. Biallelic truncating FANCM mutations cause early-onset cancer but not Fanconi anemia. Genet Med. 2018;20(4):458-463. doi: 10.1038/gim.2017.124.

S8. Broberg K, Huynh E, Schläwicke Engström K, Björk J, Albin M, et al. Association between polymorphisms in RMI1, TOP3A, and BLM and risk of cancer, a case-control study. BMC Cancer. 2009;9:140. doi: 10.1186/1471-2407-9-140.

S9. Dörk T, Peterlongo P, Mannermaa A, Bolla MK, Wang Q, Dennis J, et al. Two truncating variants in FANCC and breast cancer risk. Sci Rep. 2019;9(1):12524. doi: 10.1038/s41598-019-48804-y.

S10. Yang X, Song H, Leslie G, Engel C, Hahnen E, Auber B, et al. Ovarian and Breast Cancer Risks Associated With Pathogenic Variants in RAD51C and RAD51D. J Natl Cancer Inst. 2020;112(12):1242-1250. doi: 10.1093/jnci/djaa030.

S11. Hauke J, Horvath J, Groß E, Gehrig A, Honisch E, Hackmann K, et al. Gene panel testing of 5589 BRCA1/2-negative index patients with breast cancer in a routine diagnostic setting: results of the German Consortium for Hereditary Breast and Ovarian Cancer. Cancer Med. 2018;7(4):1349-1358. doi: 10.1002/cam4.1376.

S12. Morris MR, Astuti D, Maher ER. Perlman syndrome: overgrowth, Wilms tumor predisposition and DIS3L2. Am J Med Genet C Semin Med Genet. 2013;163C(2):106-13. doi: 10.1002/ajmg.c.31358.

S13. Takahashi M, Kawai K, Asai N. Roles of the *RET* Proto-oncogene in Cancer and Development. JMA J. 2020;3(3):175-181. doi: 10.31662/jmaj.2020-0021.

S14. Elkholi IE, Di Iorio M, Fahiminiya S, Arcand SL, Han H, Nogué C, et al. Investigating the causal role of MRE11A p.E506* in breast and ovarian cancer. Sci Rep. 2021;11(1):2409. doi: 10.1038/s41598-021-81106-w.

S15. Peck B, Bland P, Mavrommati I, Muirhead G, Cottom H, Wai PT, et al. 3D Functional Genomics Screens Identify CREBBP as a Targetable Driver in Aggressive Triple-Negative Breast Cancer. Cancer Res. 2021;81(4):847-859. doi: 10.1158/0008-5472.CAN-20-1822.

S16. Dutto I, Scalera C, Tillhon M, Ticli G, Passaniti G, Cazzalini O, et al. Mutations in CREBBP and EP300 genes affect DNA repair of oxidative damage in Rubinstein-Taybi syndrome cells. Carcinogenesis. 2020;41(3):257-266. doi: 10.1093/carcin/bgz149.

S17. Robertson JC, Jorcyk CL, Oxford JT. DICER1 Syndrome: DICER1 Mutations in Rare Cancers. Cancers (Basel). 2018;10(5):143. doi: 10.3390/cancers10050143.

S18. Swanson KD, Winter JM, Reis M, Bentires-Alj M, Greulich H, Grewal R, et al. SOS1 mutations are rare in human malignancies: implications for Noonan Syndrome patients. Genes Chromosomes Cancer. 2008;47(3):253-9. doi: 10.1002/gcc.20527.

S19. Cai D, Choi PS, Gelbard M, Meyerson M. Identification and Characterization of Oncogenic *SOS1* Mutations in Lung Adenocarcinoma. Mol Cancer Res. 2019;17(4):1002-1012. doi: 10.1158/1541-7786.MCR-18-0316.

S20. Tsukada T, Nagamura Y, Ohkura N. MEN1 gene and its mutations: basic and clinical implications. Cancer Sci. 2009 ;100(2):209-15. doi: 10.1111/j.1349-7006.2008.01034.x.

S21. Rump A, Benet-Pages A, Schubert S, Kuhlmann JD, Janavičius R, Macháčková E, et al. Identification and Functional Testing of ERCC2 Mutations in a Multi-national Cohort of Patients with Familial Breast- and Ovarian Cancer. PLoS Genet. 2016;12(8):e1006248. doi: 10.1371/journal.pgen.1006248.
